# Prophylactic Granisetron Vs Pethidine for the Prevention of Postoperative Shivering: A Randomized Control Trial

**Published:** 2009-06

**Authors:** Asif Iqbal, Ahsan Ahmed, A Rudra, Ravi G Wankhede, Saikat Sengupta, Tanmoy Das, Debasis Roy

**Affiliations:** 1,2 D.N.B.Resident, Apollo Gleneagles Hospitals, Kolkata; 3,4,5,6,7 Consultant, Apollo Gleneagles Hospitals, Kolkata

**Keywords:** Shivering, Postoperative, Granisetron, Pethidine

## Abstract

**Summary:**

Shivering-the “Big Little Problem” has an incidence of 60% in early recovery phase following general anaesthesia. A number of techniques have been tried to prevent postoperative shivering. Previous study showed that, ondansetron in higher doses reduces postoperative shivering. Therefore, this study was done to compare the efficacy of prophylactic granisetron, pethidine and placebo in preventing postoperative shivering.

Ninety patients aged 20-60yrs, ASA physical status I and II, scheduled for laparoscopic surgery under general anaesthesia were randomly allocated to receive either normal saline (Group S, n=30) as negative control, pethidine 25mg (Group P, n=30) as positive control or granisetron 40mcg.kg^−1^ (Group G, n=30) intravenously before induction. The anaesthesia was induced with fentanyl 2mcg.kg^−1^, propofol 2mg.kg^−1^ and atracurium 0.5mg.kg^−1^ and maintained with sevoflurane 1 - 1.5%. Nasopharyngeal temperature was measured throughout the procedure. An investigator, blinded to the treatment group, graded postoperative shivering in a scale of 0 to 4. (0= no shivering, 1= piloerection or peripheral vasoconstriction but no visible shivering, 2= muscle activity in only one muscle group 3= muscle activity in more than one muscle group, 4= shivering involving the whole body). Prophylaxis was regarded as ineffective if shivering was greater than grade 3 and intravenous pethidine 25 mg was administered as rescue medication.

The three groups did not differ significantly regarding patient characteristics. The numbers of patients shivering on arrival in the recovery room at 15 minutes after operation were significantly less in Group P (7%) and Group G (17%) than in Group S (60%). Groups P and G differ significantly than in Group S (*p*<0.05). However, the difference between Groups P and G was not statistically significant (*p*>0.05). The prophylactic use of granisetron (40mcg.kg^−1^) and pethidine(25mg) intravenous were found to be effective in preventing postoperative shivering.

## Introduction

Post anaesthetic shivering is one of the most frequent problems in the early recovery phase following general anaesthesia^1^,^2^. Considering clinical importance and frequency, postanaesthetic shivering was ranked as the sixth most important problem of current clinical anaesthesiology among 33 low morbidity clinical outcomes.^3^ Previous studies have found that shivering occurs in the postoperative period in up to 60% of patients^1^,^2^,^4^ and varies according to age, sex, drugs used for anaesthesia and the duration for the surgery^4^.

Postanaesthetic shivering is not only distressing to patients, but can lead to physiological changes such as increased tissue oxygen consumption and carbon dioxide production, resulting in raised minute ventilation and cardiac output^5^. Moreover, the elderly with limited cardiopulmonary reserve may suffer form lactic acidosis, mixed venous oxygen desaturation, and hypoxemia^4^–^6^. A number of pharmacological intervention have been studied for the treatment and prophylaxis of shivering, including clonidine, ketamine, doxapram, tramadol, pethidine and other opioids^6^–^9^. Among the pharmacological agents, pethidine has been shown to be one of the most effective treatment^10^,^11^. Although its mechanism of action is not completely understood, it probably acts directly on the thermoregulatory centre^12^ or via opioid receptors^13^. Serotonin (5-Hydroxytryptamine), a biological amine found in the brain and spinal cord, has a role in neurotransmission and studies suggest that the serotonergic system has a role in control of postanaesthetic shivering^9^. Granisetron, 5-HT_3_ receptor antagonist, has been shown to be effective in the prevention of emetic symptoms^14^,^15^. The best of our knowledge, there is no study in India regarding the use of granisetron as a prophylactic agent against postoperative shivering. The aim of the study was to compare the efficacy of prophylactic granisetron on postanaesthetic shivering in comparison to pethidine an agent which is known to be effective in the treatment and prevention of postanaesthetic shivering^10^.

## Methods

Following institutional review board approval and after obtaining written informed consent, a prospective, randomized, double-blind, placebo-controlled study was undertaken. The power of the study was calculated based on the number of the patients who shivered, setting a significant level of *p* = 0.05, it was calculated that a group size of 30 patients allowed detection of a difference between groups with a power of 80%. Therefore, we took 90 patients aged 20 yr to 60yr, ASA physical status I and II, undergoing laparoscopic surgery. Patients with cardiopulmonary disease, psychological disorder, and with body temperature more than 38°C or less than 36.5°C were excluded from the study. Procedures which might require administration of blood and blood products and anticipated duration more than 180 min were also excluded from the study.

The patients were randomly (enveloped randomization) allocated to receive normal saline (Group S, n = 30) as negative control, pethidine 25mg (Group P, n=30) as positive control or granisetron 40mcg.kg^−1^ (Group G, n = 30) as study agent intravenously before induction of anaesthesia.

The prepared drug was diluted to a volume of 5 ml and presented as coded syringes by anaesthesiologists who were not involved in the management of patients.


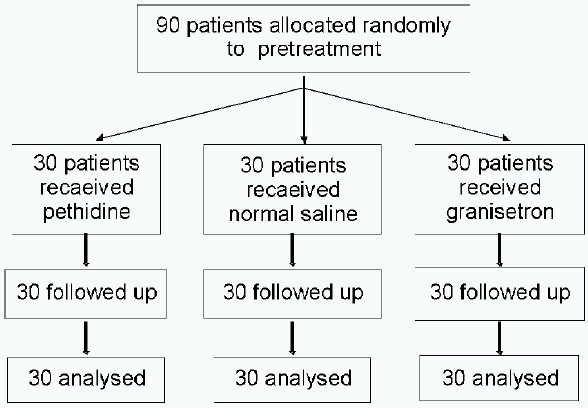


The anaesthetic management of the patients were standardized. Heart rate, non-invasive blood pressure, oxygen saturation and end tidal carbon dioxide recorded during the surgery. Nasopharyngeal temperature (as we do not have tympanic probe) was measured as core body temperature immediately after induction of anaesthesia and continued till the completion of surgery. Operation room temperature and recovery room temperature standardized by centrally air-conditioning with laminar flow and hepafilter.

Anaesthesia was induced with fentanyl 2mcg.kg^−1^, propofol 2mg.kg^−1^ and atracurium 0.5mg.kg^−1^ was given to facilitate orotracheal intubation. Anaesthesia was maintained with 1 - 1.5% sevoflurane in oxygen and air, ventilator was adjusted to maintain end tidal carbon dioxide between 4.6 and 5.2 kPa throughout the procedure. Muscle relaxation for pneumoperitonium and surgical procedure was provided with additional doses of atracurium. During laparoscopy, intra-abdominal pressure was maintained at 1.3 to 1.8 kPa by carbon dioxide insufflator. Residual neuromuscular blockade was antagonized with neostigmine 0.05 mg.kg^−1^ and glycopyrrolate 8-10 mcg.kg^−1^. When the patient respiratory efforts were adequate and he or she responded to verbal commands, the trachea was extubated. The type and duration of anaesthesia and surgery were recorded.

In the recovery room, all patients were monitored, received oxygen through facemask and were covered with cotton blanket. An anaesthesiologist unaware of the study drug observed the patients for shivering, pain, nausea and vomiting. Heart rate, non-invasive blood pressure, oxygen saturation and nasopharyngeal temperature were measured and recorded on admission to the recovery room at 15 minute. The shivering was graded using a scale similar to that validated by Tsai and Chu^16^ (Table-1). Any possible side effects of the study drug (i.e. nausea, vomiting, hypotension, tachycardia, dry mouth, and dizziness) were recorded. Patient with nausea and vomiting were treated with metoclopramide 10mg. If the patient shivered according to at least grade 3 the prophylaxis was regarded as ineffective and intravenous pethidine 25 mg was administered as rescue agent^17^.

**Table 1:** Classification of shivering

Score/Grade	Clinical Signs
0	No shivering
1	Piloerection but no visible shivering
2	Muscular activity only one muscle group
3	Muscular activity more than one muscle group but not generalized
4	Shivering involve the whole body

The incidence of shivering and side effects were compared using the Chi square test. The results were reported as mean ± SD. *p* < 0.05 was considered statistically significant.

## Results

The three groups were comparable regarding distribution of age, weight, height, gender, duration of anaesthesia, duration of operation and ASA physical status Table 2.

**Table 2:** Patient characteristics of the three treatment groups. Data are given as mean±SD (range), mean (SD) or absolute numbers

	Group S (n = 30)	Group P (n = 30)	Group G (n = 30)
Age (yr)	43 (20-60)	45 (20-60)	45 (20-60)
Female/Male	23/7	24/6	24/6
Weight (kg)	60(6)	65(10)	65(9)
Height (cm)	164(6)	164(8)	162(9)
ASA I/II	25/5	26/4	26/4

The number of patients with postoperative shivering on arrival in the recovery room, 15 minutes after arrival, were significantly less in Group G and Group P than in Group S (*p*<0.05) in Table 3. There was no statistically significant difference between Group P and G (*p* > 0.05) in Table 3. In Group S, 18 patients shivered at grade=3 and were subsequently treated with pethidine 25 mg intravenously as rescue agent. However, in Group G and Group P only 6 and 2 patients reached grade 3 shivering respectively (Table 3). However, there were no significant differences in the core temperature amongst the patients before and after the anaesthesia (Table 4).

**Table 3:** Number of patients with different grades of shivering in the three treatment groups after arrival in recovery room in 15 minute.

		Grade 0/1/2/3			
Shivering score	Group S	Group P	Group G	
0	9	26*	21*	*p*<0.05
1	1	1	2	
2	2	1	1	
3	18†	2	6	*p*<0.05
4	0		0	

* *p*<0.05 compared with the other groups, using the Chi-square test,

† *p*<0.05 compared with the other groups, using the Chi-square test

**Table 4:** Variation in core temperature (°c) [mean±SD]

	OR Temperature		Core Temperature(°C)		
		Group S	Group P	Group G	*p* value
Pre-operative	21-22°C	36.7±0.32	36.6±0.41	36.7±0.39	>0.05
Post-operative	21-22°C	35.61±0.25	35.48±0.18	35.59±0.27	>0.05
Difference		1.09±0.07	1.12±0.23	1.11±0.12	>0.05

* *p*<0.05 compared with the other groups, using the Chi-square test,

Five patients in Group S and seven patients in Group P had nausea and vomiting (*p*>0.05) but no patients of Group G complained of nausea and vomiting. None of the patients had episodes of oxygen desaturation or respiratory depression during the study. None of the patient in the study groups had cardiovascular complication.

## Discussion

In this study, we found that granisetron 40mcg.kg^−1^ was as effective as pethidine 25mg in preventing shivering related to general anaesthesia.

During postoperative period, shivering is a frequent and understandable complication of general anaesthesia^18^ and the incidence has been shown around 56%-66% following general anaesthesia^1^,^2^,^4^. In our study, we also found 60% of patients in Control group had shivering after general anaesthesia. Shivering increase metabolic activity and oxygen consumption. It may also cause arterial hypoxia and lactic acidosis. Furthermore, it may interfere with the monitoring of an electrocardiogram^16^,^19^. All of these make the prevention of shivering important especially in elderly patients with a low cardiopulmonary reserve^16^.

It has been mentioned that hypothermia may cause postanaesthetic shivering by alteration of thermoregulatory mechanism^12^. However, no relationship has been shown between axillary temperature and occurrence of shivering.^12^ In our study there were no significant differences in nasopharyngeal temperature among the groups.

A number of factors including age, duration of surgery, temperature of the operating room, and infusion solution, are risk factors for hypothermia and shiverring^13^. For this reason, in our study patients over the age of 60 years were excluded^13^. The temperature of operation room was maintained at 24°C and infusions of cold crystalloid solution were avoided.

Various drugs have been used to treat or prevent postoperative shivering but the ideal treatment has not yet been found. 5-hydroxytryptamine may influence both heat production and heat loss pathways^13^. Ondansetron (4 and 8 mg) and dolasetron (1mg.kg^−1^), 5-HT_3_ antagonists have been effectively used in treatment of postoperative shivering^4^,^20^. Powell and Colleagues^4^ reported that after general anaesthesia, shivering was determined in 57%, 33% and 15% of patients in control, ondansetron 4mg and 8mg respectively. Similarly, Bock and colleagues^20^ mentioned in their study report that dolasetron 1mg.kg^−1^ decreases the incidence of shivering from 62% to 27%. The incidence of shivering (27%) in patients who received dolasetron in their study was more than the incidence of shivering in the patients those who received granisetron in our study (17%), however, the difference is not statistically significant.

Pethidine has been shown to be one of the most effective treatments to prevent postoperative shivering^10^,^11^. In our study, two patients shivered after prophylactic pethidine, however the mechanism of pethidine to reduce shivering is not clear. The study using naloxone indicated that pethidine may act via *k* receptor than μ opioid receptors to prevent shivering. The anti-shivering action of pethidine was inhibited by high dose naloxone, which blocks both μ and *k* receptors, but not by low dose of naloxone which block only μ receptors^10^. A disadvantage of pethidine is that it can cause respiratory depression in the presence of previously administered opioids or anaesthetics. Moreover, nausea and vomiting are also important side effect of pethidine.

In conclusion, pethidine can cause some side effects such as respiratory depression, hypotension, and postoperative nausea and vomiting. Therefore, prophylactic granisetron 40mcg.kg^−1^ intravenously may be administered without causing the adverse effect in patient with high expectancy of shivering and postoperative nausea and vomiting ranked sixth and second in morbidity clinical outcome.^3^
